# Energy-Rich Molecules and Group Transfer Potentials in Energetic Coupling Reactions

**DOI:** 10.3390/molecules31020242

**Published:** 2026-01-11

**Authors:** Lucien Bettendorff, Pierre Wins

**Affiliations:** Laboratory of Neurophysiology, GIGA Institute, University of Liège, 4000 Liège, Belgium; winspierre9@gmail.com

**Keywords:** energy coupling, energy metabolism, bond energies, energy rich molecules, ATP, transfer potential, phosphoryl transfer

## Abstract

The concept of energy-rich molecules is central to metabolic activity and the coupling of catabolic and anabolic processes. Here, we use the term “energy-rich” only in the (bio)chemical sense, i.e., for molecules containing particularly weak bonds that when exchanged for stronger bonds results in a release of energy (generally ≥ 20 kJ mol^−1^). The typical energy-rich molecules are nucleoside triphosphates (NTPs), thioesters, and dioxygen. It must be emphasized that the number of bonds is conserved in biochemical reactions, so that the difference in free energy between substrates and products only depends on the difference in bond energies. It is evident that using the term “energy-rich” for molecules with weak bonds is subject to misinterpretation. Therefore, some authors suggested to replace this term by molecule of high group transfer potential. This is justified for NTPs and thioesters, which have a high transfer potential for, respectively, phosphoryl or acyl groups, but not for dioxygen. Therefore, the concepts of energy-richness and group transfer potential should be treated as different and only be used within specific contexts. We discuss how these two notions can be used to understand the coupling mechanisms in biochemical processes as well as the interplay between thioesters, redox coupling, and phosphate transfer reactions.

## 1. Introduction

A conspicuous feature of living cells is their extraordinary efficiency as energy transducers. Indeed, biological systems have evolved highly efficacious mechanisms for using the energy obtained from sunlight or the oxidation of organic fuels to create and maintain their highly ordered structures and to render possible all the energy-consuming processes necessary for cell survival, growth, and replication.

Such mechanisms, referred to as energetic coupling, are essential for maintaining living cells in a steady state far from thermodynamic equilibrium with minimal entropy production. They are found only in biological systems and are the basis for the high energetic efficiency of biochemical processes (compared to the lower efficiency of, e.g., heat engines).

**Figure 1:**
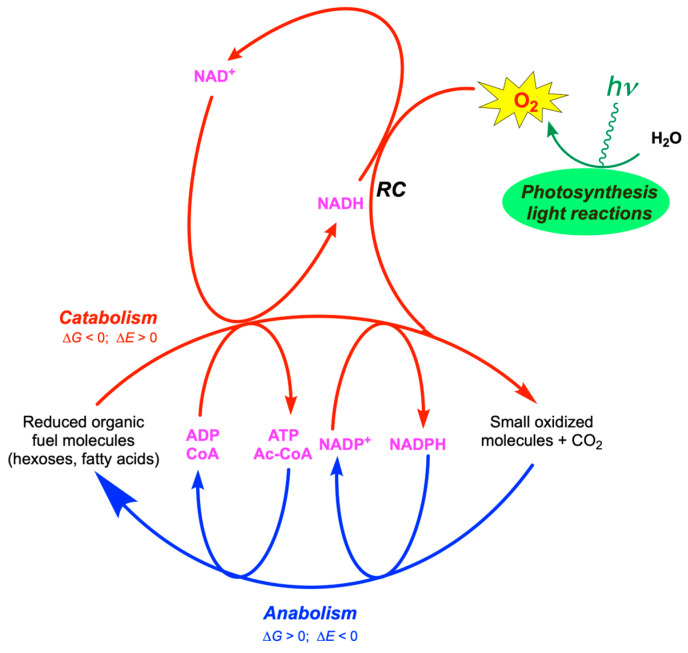
Basic energy coupling events in living systems: photosynthesis (green), catabolism (red), anabolism (blue), and coupling agents (magenta). Catabolic pathways are globally exergonic (Δ*G* < 0) and oxidative (with respect to carbon) in nature (Δ*E* > 0), while anabolic pathways are endergonic (Δ*G* > 0) and reducing (Δ*E* < 0). While ATP, acetyl-CoA (Ac-CoA), and NADPH couple catabolism with anabolism, NADH operates only within the catabolic block to supply the respiratory chain with reducing power. (*RC*, respiratory chain; *E*, redox potential).

Metabolism is the set of interconnected life-sustaining reactions that take place in all biological systems. More specifically, energy metabolism is a sequence of reactions that result in the production of intermediate molecules such as ATP and NAD(P)H, that we call coupling agents, as they are required to couple catabolic (energy-yielding) pathways to anabolic (energy-consuming) processes (Figure 1). Metabolism could not exist in the absence of coupled reactions.

Most living cells obtain the energy they need by oxidizing organic fuel molecules (ultimately produced by photosynthesis) to form water, CO_2_, and other products which are recycled in the environment [1].

**Figure 2:**
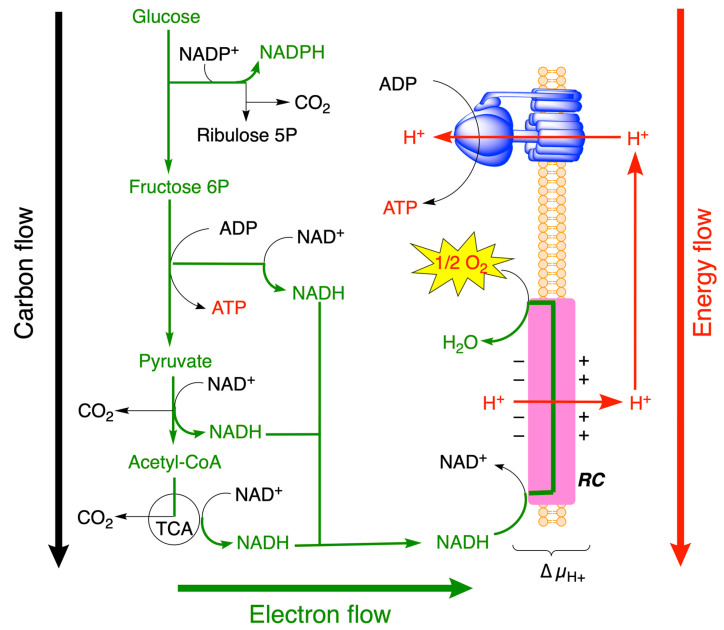
Catabolic fluxes of matter (carbon), energy, and electrons through the central energy metabolic pathways (glycolysis, tricarboxylic acid (TCA) cycle, and pentose phosphate pathway). For simplification, no subcellular compartmentations are indicated, and the scheme may concern any prokaryotic or eukaryotic organism. The driving forces (Δ*G*′) depend on differences in concentrations (*Q*) and the structural rearrangement between initial and final reactants (ΔG′°) and redox potential (Δ*E*′). (modified from [1]).

Fuel molecules such as glucose are degraded through a sequence of energy-releasing processes, i.e., the glycolytic, pentose phosphates, and tricarboxylic acid (TCA) pathways (Figure 2). The main molecules coupling catabolism and anabolism are NADPH and ATP. NADPH is produced through the reaction catalyzed by the oxidative portion of the pentose phosphate shunt (glucose 6-phosphate and 6-phosphogluconate dehydrogenases). An anabolic process such as the synthesis of fatty acid will use the reducing power of NADPH and the chemical energy of ATP (Figure 2).

For glycolysis, the global reaction is [2]:

glucose + 2 ADP + 2 P_i_ + 2 NAD^+^ → 2 pyruvate + 2 ATP + 2 H_2_O + 2 NADH + 4 H^+^  ΔG′° = −73.7 kJ mol^−1^

Two molecules of ATP are produced by reactions catalyzed by phosphoglycerate kinase and pyruvate kinase (Figure 2).

In further catabolic reactions catalyzed by pyruvate dehydrogenase and enzymes of the TCA cycle, pyruvate is decarboxylated and oxidized by NAD^+^ and FAD, yielding NADH, FADH_2_, and CO_2_ [2,3]:

Pyruvate + NAD^+^ + CoA → Acetyl-CoA +NADH + H^+^ + CO_2_  ΔG′° = −33 kJ mol^−1^

Acetyl-CoA + 3 NAD^+^ + FAD + GDP + P_i_ +2 H_2_O → CoA-SH + 3 NADH + 3 H^+^ + FADH2 + GTP + 2 CO_2_  ΔG′° = −50.3 kJ mol^−1^

Note that we use, whenever possible, the biochemical recommendations with transformed variables defined at pH 7 [4] (Section 3.1).

In turn, NADH and FADH_2_ are oxidized by O_2_ in the respiratory chain, the latter being coupled to ATP synthesis by a chemiosmotic mechanism. Hence, in contrast to NADPH which is involved in the transfer of reducing equivalents from catabolism to power anabolism, NADH is involved in the transfer of reducing equivalents within the catabolic block (Figure 1 and Figure 2).

If we consider the global reaction of glucose oxidation through glycolysis, TCA cycle, and oxidative phosphorylation (C_6_H_12_O_6_ + 6 O_2_ + 32 ADP + 32 P_i_ → 6 CO_2_ + 38 H_2_O + 32 ATP), a maximum of 32 molecules of ATP are generated, close to an energetic yield of 40% [2,5]. The carbon from reduced organic molecules ends up in CO_2_, its most oxidized form.

ATP is often called the “energy currency” of the cell: it is central to most biochemical energy-transferring processes as an intermediate between energy-yielding and energy-requiring reactions, just as NADH and NADPH are intermediates in redox coupling.

As discussed previously [1], the transformation of carbonated molecules (the carbon molecules of the partially reduced molecule glucose ending up in the oxidized CO_2_ molecule) can be equated to a flow of matter (actually carbon) resulting from differences in concentrations (*Q*) and the structural arrangements between initial and final reactants (Δ*G*′°) with Δ*G*′ = Δ*G*′° + RT ln *Q*, as well as a flow of electrons arising from differences in redox potentials (Δ*E*′ = Δ*E*′° + $\frac{RT}{zF}$ ln *Q*) with Δ*G*′ = −*zF*Δ*E*′. Hence, the differences in nature and concentrations of the reactants and products of the metabolic activity of living cells are responsible for a flow of free energy (Δ*G*′).

In this review, we will discuss the concept of chemical energy and the question of how it is transferred and conserved during energetic coupling processes and how the notions of high-energy molecule and high group transfer potential can be reconciled.

## 2. Energy Changes in Chemical Reactions

### 2.1. Chemical Energy and Molecular Bonding

In the first place, it is important to define the term energy in the chemical context. Energy by itself is a quantity difficult to grasp because it is invisible. Generally, the only manifestation of energy we see is the change in energy when a system proceeds from one state to another. Chemists tend to define chemical energy as the energy released or absorbed when a substance undergoes a chemical reaction, i.e., a process where some bonds are broken and new bonds are formed, creating a new substance [6].

The question that immediately arises is why do atoms come together? What determines the possibility (or impossibility) of the assembly of two atoms?

We will address the problem by considering the simple example of two hydrogen atoms that, at the beginning, are far from each other. Such a system has a certain potential energy due to the attraction between the nucleus of one and the electron cloud of the other. This potential energy decreases as the two atoms become closer. But when the distance between the two atoms becomes very small (of the order of magnitude of the atomic diameter), the situation is reversed: the force of attraction gives way to a repulsion between the two atoms which increases very quickly if they continue to move closer.

In between the distances at which atoms attract each other and those at which they repel each other, there is a position of equilibrium at which the two effects compensate and cancel each other out. The potential energy of the system is minimal and atoms can oscillate around their equilibrium distance, but they are always brought back to it [7].

Since the two atoms cannot separate spontaneously and it takes work to separate them, they are said to be bonded. The H_2_ molecule exists because its potential energy is lower than that of the separated atoms.

**Figure 3:**
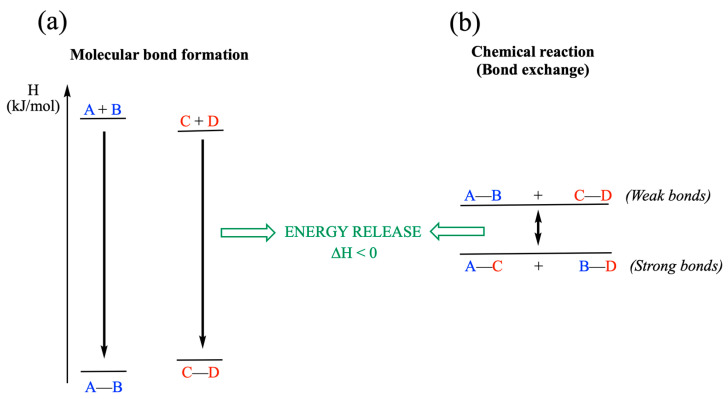
Molecular bond formation and chemical reaction (bond exchange). (**a**) Molecular bond formation is always an exothermic process (Δ*H* < 0). (**b**) Bond exchange in the exothermic chemical reaction A–B + C–D ⇄ A–C + B–D. The replacement of weak bonds by strong bonds leads to a release of energy. Note that the relatively small Δ*H* involved in many biochemical bond exchange reactions (often 10–15 kJ mol^−1^) can be overcome by coupling to exergonic reactions.

The difference in potential energy between the “separate atoms state” and the “bonded atoms state” is called the bond dissociation energy. It represents both the energy released when the bond forms spontaneously (for instance A + B → A–B) and the energy that must be provided to break it (Figure 3a).

Chemical bond energies are estimated by the enthalpy of formation Δ_f_*H*° from the elements in the standard state. As shown in Table 1, most bonds involving only one electron doublet (single bonds) have Δ_f_*H*° values that are rather close to each other (300–450 kJ mol^−1^). In contrast, double bonds such a C=O are generally stronger (Δ_f_*H*° is about twice times more negative), with the notable exception of the dioxygen molecule which has a low Δ_f_*H*° (see below).

### 2.2. Energetics of Chemical Reactions

In a second step, let us now consider, not the reaction between two atoms, but between two molecules that exchange groups (Figure 3b):

A—B + C—D ⇄ A—C + B—D

It is the connectivity of the bonds that changes, and this difference is the driving force of the reaction. Moreover, while bond formation is always exothermic, bond exchange reactions can be either exothermic (weak bonds are exchanged for stronger bonds) or endothermic (strong bonds are exchanged for weaker bonds).

The difference in energy between reagents and products is only the result of different bonding energies (i.e., different molecular rearrangement) and this difference is, in most cases, an order of magnitude smaller than a bond energy. Hence, the replacement of a C—C bond by a C—O bond would result in the release of only 5 kJ mol^−1^ (Table 1). This value is, of course, only an approximation as the real bond energy depends on the molecule and the molecular environment of the bond to be broken.

The so-called “energy-rich” molecules have at least one covalent bond that is relatively weak (low energy) and can be replaced by a stronger bond (more energetic). Thus, the Δ*G*′° of reaction becomes negative [8,12].

Let us now consider a fundamental reaction of catabolism, i.e., glucose oxidation:

C_6_H_12_O_6_ + 6 O_2_ → 6 CO_2_ + 6 H_2_O  Δ*G*′° = −2870 kJ mol^−1^

Once again, the simple inspection of the structural formulas of reactants and products shows that the number of molecular bonds does not change (which is the norm in biochemical reactions). Glucose combustion is strongly exergonic but, in vivo, the oxidation of glucose is a sequence of many individual reactions with much lower ΔG′° values.

It is because of the relatively weaker energies involved in biochemical reactions, compared to bond energies, that energetic coupling is possible under mild conditions (temperature, pH), compatible with life.

## 3. The Concept of Energy-Rich Molecules and Their Role in Energy Metabolism

A fundamental question in bioenergetics is how living cells store the free energy required for their survival and various activities. It has traditionally been assumed that the energy is stored primarily in fuel molecules such as sugars and fatty acids. However, as will be discussed below, neither hexoses nor fatty acids can be considered as “rich in energy”. Indeed, energy is released only when these molecules react with oxygen [8,13,14].

The so-called energy-rich molecules in living systems are limited to only three main groups: several phosphorylated compounds (nucleoside triphosphates, acyl phosphates…), thioesters (acetyl-CoA…), and, more unexpectedly, O_2_ [15].

### 3.1. ATP and Other Energy-Rich Phosphorylated Compounds

Before proceeding, let us consider the chemical equation for ATP hydrolysis written as recommended by the IUBMB/IUPAC [4]:

ATP^4−^ (*aq*) + 2 H_2_O (l) ⇄ ADP^3−^ (*aq*) + HPO_4_^2−^ (*aq*) + H_3_O^+^ (*aq*)

**Figure 4:**
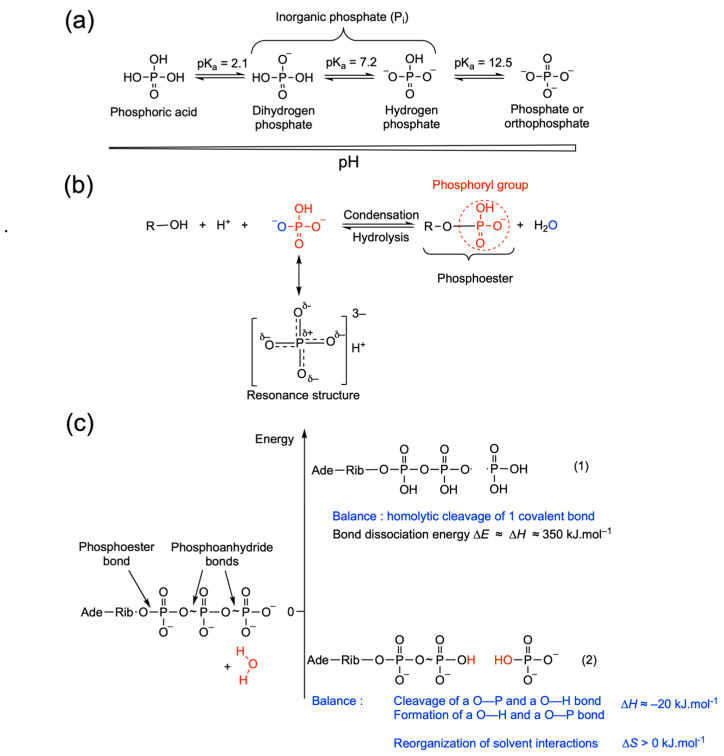
Relations between bond dissociation enthalpies and energy of hydrolysis. (**a**) Ionization of the phosphate anion as a function of pH. At physiological pH, the ratio H_2_PO_4_^−^/HPO_4_^2−^ is close to 1. (**b**) During hydrolysis, a phosphoryl group is transferred to water to form inorganic phosphate. The reverse condensation reaction invariably involves the abduction of a hydrogen (H^+^) and hydroxyl (OH) group to form water (H_2_O). Note that the usual way of drawing the phosphate ion or the phosphoryl group with one double bond is misleading, as all P–O bonds share some double bond character, and the negative charge(s) is (are) equally distributed among all non-esterified oxygen atoms, as shown by the resonance structure for HPO_4_^2−^. (**c**) The first reaction (1) is a homolytic cleavage of the γ-phosphoryl group and requires a strong input of energy. Strictly speaking the bond dissociation energy (Δ*E*) is defined at 0 K, while the enthalpy change is measured under normal standard conditions. The small difference can, however, be neglected here. The second reaction (2) corresponds to the hydrolytic cleavage with transfer of the γ-phosphoryl to water. This reaction releases energy Δ*H*° ≈ Δ*H* = −20 kJ mol^−1^ (measured at pH 8 in Tris buffer with 0.6 M KCl [16]). As in the previous example, the number of molecular bonds does not change, two bonds are broken, and two bonds are formed.

This equation is written, as any chemical reaction, in order for H^+^ and charges to be balanced. However, it is essential to realize that phosphate and phosphorylated compounds in general are a mixture of different species differing by their state of ionization and depending on pH (Figure 4a). As biochemists most often work at fixed pH (pH = 7, [H^+^] = 10^−7^ M), and H^+^ and charge do not balance, it is recommended to not explicitly show charges in the biochemical equation [4]:

ATP + H_2_O ⇄ ADP + P_i_   ΔG′° = −30.5 kJ mol^−1^

This leads to the use of transformed standard free Gibbs energies of reaction (Δ*G*′°) defined at pH = 7 and where ATP refers to an equilibrium mixture of ATP^4−^, HATP^3-^, and H_2_ATP^2−^, and P_i_ refers to the equilibrium mixture of H_2_PO_4_^-^ and HPO_4_^2−^ at pH 7.

Hence, biochemists commonly use the term inorganic phosphate (P_i_) to designate the phosphate anion in biological fluids. On the other hand, a phosphoryl group (Figure 4b) is the functional group transferred in biological reactions (kinase reactions for instance). When the phosphoryl group is transferred to water (a reaction catalyzed by phosphatases), it becomes a phosphate anion (P_i_).

ATP is the canonical “high-energy molecule” of the biological world. It has traditionally been assumed that this is because it contains two so-called “high-energy” phosphoanhydride bonds (Figure 4c). This old misconception arose from the well-known observation that reactions where those bonds are broken (e.g., ATP hydrolysis) are indeed rather strongly exergonic.

Actually, phosphoanhydride bonds are by no means rich in energy. They are weak bonds that are easily broken in the presence of a nucleophilic phosphate acceptor such as H_2_O. In the absence of water, those anhydride bonds remain quite stable, even if they are weak. Only a hydrolytic cleavage (which is a group transfer displacement reaction) releases a relatively high amount of free energy. Even if molecules such as ATP and acyl phosphates are referred to as energy-rich, it must be understood that they have a high potential of energy transfer, not that they have a higher content of chemical energy than other molecules.

The high potential of energy transfer of ATP and other compounds with phosphoanhydride or acyl phosphate bonds has led Lipmann (1941) [12] to propose the symbol “~” (squiggle) for the bonds that are broken when those compounds are hydrolyzed or when they transfer phosphoryl groups to other compounds. However, as pointed out by Atkinson [17], the squiggle formulation has been misunderstood and perverted in many ways. In fact, hardly has an important notion in biochemistry been so mistreated.

Generations of students in all fields of biology have been taught that the energy needed for vital processes is provided by the breaking of “high energy” bonds ([6,18,19] to name only a few). This is, of course, not consistent with what these same students have been learning about chemical bonds in first year chemistry courses, i.e., that energy is required, not released when bonds are broken. Thus, it must be understood that free energy is released only when the phosphoryl group is transferred to a suitable acceptor such as water, not when the phosphoanhydride bond is broken (Figure 4c).

Although modern biochemistry texts no longer refer to high-energy bonds, the squiggle bond concept survived because it was—or seemed to be—very useful: it led biochemists to think stoichiometrically about metabolism and to recognize the quantization of metabolic energy [17].

Thus, using the expression “cleavage of ATP” (or ATP splitting) remains tricky and should be used only if it refers to a precise catalytic step, or when referring to the splitting of a precise bond, for instance the P—O bond, rather than the complete reaction of hydrolysis of ATP.

### 3.2. What Makes the Hydrolysis of Phosphoanhydride Bonds in Atp and Other Nucleotides So Particular?

This question is still debated, though it is generally recognized that at least three factors are important: electrostatic repulsion of oxygen atoms in ATP, and resonance stabilization and better solvation of hydrolysis products versus reactants [6,20]. In addition, anomeric effects may participate [21].

First, at physiological pH, the linear polyphosphate portion of ATP has four negative charges that are very close and repel each other. When the terminal phosphate is released, part of this electrostatic stress is relieved.

Second, the enhanced solvation of products relative to the reactants is another important factor.

The third major factor contributing to the negative value of Δ*G*′° for ATP hydrolysis is the fact that the two products ADP and P_i_ undergo stabilization as resonance hybrids: Such resonance stabilization of the hydrolysis products is a major reason for the large negative value for the standard free energy of hydrolysis of ATP. This is also the case for the hydrolysis of other energy-rich molecules such as acetyl phosphate. As can be seen from Table 2, those molecules that have a lower energy of hydrolysis than ATP yield P_i_ and a non-resonance stabilized product (glucose or adenosine). Molecules with a higher energy of hydrolysis than ATP yield P_i_ and a resonance-stabilized product. In contrast, the coupling of phosphates to the 5′-OH end of adenosine forms a relatively stable phosphoester bond. Here, the repulsive forces between oxygen atoms do not exist and the hydrolysis of this bond releases much less energy (Table 2).

The intermediate position of ATP on the thermodynamic scale (Table 2) allows it to be an ideal coupling agent between energy supplying and energy utilizing reactions. The ATP/ADP couple forms a “shuttle” for phosphoryl groups and it is therefore not surprising that the terminal phosphoryl group of ATP undergoes very rapid turnover in cells with a half-life < 1 s^−1^. Obviously, “energy-rich” compounds such as ATP play an essential role in maintaining the non-equilibrium state of living systems and in driving thermodynamically unfavorable processes.

The above arguments are true for all NTPs (GTP, UTP, TTP, and CTP), which are all energy-rich molecules with similar properties than ATP. They are remnants from an RNA-world, and in addition to being the building blocks for nucleic acids, all serve as energy currency in some reactions, but only ATP was selected as the universal currency [22]. We can only guess that the reason is that adenosine is relatively easily formed from prebiotic precursors such as HCN [23]. Ribose and deoxyribose probably have the right size to form stable nucleic acids and a stable double helix, a prerequisite for the depositary of the genetic information [24].

**Table 2:** Standard free energies of hydrolysis (Δ_h_*G*′°) and phosphoryl group transfer potentials (−Δ_h_G′°) of some phosphorylated molecules of major importance in biochemistry at pH 7 (298 K).

Donor	Δ_h_*G*′° ^1^ (kJ mol^−1^)	Phosphoryl Group Transfer Potential (−Δ_h_*G*′°)
Phosphoenolpyruvate	−61.9	61.9
1,3-Bisphosphoglycerate	−49.4	49.4
ATP (to AMP)	−45.6 ^2^	^3^
Acetyl phosphate	−43.1	43.1
Creatine phosphate	−43.1	43.1
Pyrophosphate (PP_i_)	−33.0 ^4^	33.0
ATP (to ADP)	−30.5	30.5
ADP (to AMP)	−30.5	30.5
Glucose 1-phosphate	−20.9	20.9
5-AMP	−14.2	14.2
Glucose 6-phosphate	−13.8	13.8
Glycerol 3-phosphate	−9.2	9.2

^1^ From [25]; ^2^ from [26]; ^3^ diphosphoryl group transfer; ^4^ some data suggest a Δ_h_*G*′° of −15 to −33 kJ mol^−1^ under physiological conditions [27].

Phosphates may also condense into di-, tri-, and polyphosphates. Such compounds are still present in all organisms and may have played a role as an energy source in prebiotic chemistry and even in early living organisms [28]. In contrast to carbonic acid, for instance, phosphate is stable with no strong tendency to lose H_2_O. Only the condensation of two phosphate ions proceeds through the elimination of an intramolecular water molecule. But NTPs present the advantage over inorganic polyphosphates that the base moiety adds a handle to the phosphate part, allowing specific recognition by enzymes. Incidentally, the blocking of one end of the phosphate part also stops further addition of the phosphate group at this terminal, preventing its extension to insoluble polyphosphates [29].

### 3.3. Thioesters Are Common Intermediates Between Catabolism and Anabolism

Thioesters, in which a sulfur atom replaces the usual oxygen in the ester bond, have a much larger negative energy of hydrolysis than O-esters [30]. This is related to the fact that thioesters undergo much less resonance stabilization than the O-esters: the overlapping between 2*p* and the larger 3*p* (sulfur) orbitals is less than between two 2*p* orbitals [31]. Those factors result in the large negative Δ*G*′° for the hydrolysis of a thioester:

R—C(=O)—S—R’ + H_2_O → R—C(=O)—OH + R′—SH

The Δ*G*′° for the hydrolysis for acetyl-CoA (−31 kJ mol^−1^) is close to the Δ*G*′° for ATP hydrolysis, and thioesters may thus be considered as “energy-rich” compounds. They play a key role as shuttles between electron or hydrogen transfer reactions (such as the oxidation of an aldehyde by NAD^+^) and a group transfer reaction (such as the phosphorylation of ADP). Thus, thioesters have the remarkable property to allow the direct formation of a chemical bond using the energy released by an electron transfer [32].

### 3.4. Dioxygen Is an Energy-Rich Molecule

It is well known that the ultimate energy source for living systems is solar energy. However, most living cells are devoid of photosystems and even a photosynthetic bacterium has to store chemical energy to survive during dark hours.

It has been traditionally assumed (and stated in most biology and biochemistry textbooks, see for instance Nelson and Cox 2000, page 8 [30] or Stryer page 463 [33]) that in most cell types, energy is stored in fuel molecules such as sugars and lipids (for a more exhaustive discussion see [15]). This view is no longer tenable [8,15]. Indeed, already over a century ago, W.M. Thornton noted that the heat released in combustion reactions depends directly on the number of oxygen molecules involved, regardless of the nature of the organic compound [13]. Much later, Hilton M Weiss [14] correctly noted that organic fuels are among the most stable organic molecules found in nature. Indeed, fossil fuels, such as alkanes, stay in the Earth’s crust for hundreds of millions of years. These compounds, made up of solid bonds, have little tendency to combine with other molecules.

In order to illustrate this concept, let us consider the apparent energy content of a biological relevant fuel molecule such as glucose. It is well known that much energy is released by the combustion of glucose:

C_6_H_12_O_6_ + 6 O_2_ → 6 CO_2_ + 6 H_2_O      Δ*G*′° = −2870 kJ mol^−1^

In contrast, glucose decomposition to carbon and water in the absence of O_2_ releases much less energy [15]:

C_6_H_12_O_6_ → 6 C + 6 H_2_O   Δ*G*° = −511 kJ mol^−1^

Note that we use the standard value at pH 0 (Δ*G*°), when the transformed value at pH 7 (Δ*G*′°) is not available. The difference is generally small, provided H^+^ does not appear in the reaction.

Likewise, alcoholic fermentation is only weakly exergonic even though two strongly bonded molecules (CO_2_) are formed:

C_6_H_12_O_6_ → 2 C_2_H_5_OH + 2 CO_2_.   ΔG′° = −236 kJ mol^−1^

As emphasized by Schmidt-Rohr [8,15], the important factor for energy release is the reaction with O_2_ rather than mere decomposition of the glucose molecule. Indeed, when organic molecules react with O_2_, invariably, a lot of energy is released. This is also the case for ethanol combustion, which is nearly as exergonic as glucose oxidation:

2 C_2_H_5_OH + 6 O_2_ → 4 CO_2_ + 6 H_2_O    Δ*G*° = −2638 kJ mol^−1^

Hence, more O_2_ in the reaction results in the release of more energy, almost regardless of the nature of the fuel molecules or reaction products. The obvious interpretation of these observations is that a lot of chemical energy is stored in the O_2_ molecule rather than in glucose [15].

In view of the above considerations, we can now explain why the combustion of glucose releases 2870 kJ mol^−1^, nearly ten times the energy of an electron doublet bond, while the reaction is just a rearrangement of molecular bonds (Section 2.2). In fact, this is mainly due to the properties of the dioxygen molecule with a particularly weak bonding energy [8,34].

Indeed, although double bonds are generally significantly stronger than single bonds, the O=O double bond in the oxygen molecule is only slightly stronger than the H–H bond in the hydrogen molecule, whereas the C=O bonds, for instance, are much stronger (Table 1). These special properties of the oxygen molecule make it a particularly powerful oxidant, able to react with most of the reducing molecules present in living cells. It is therefore not surprising that O_2_ is the most used substrate for respiration, which is the main way of energy production in living systems.

**Figure 5:**
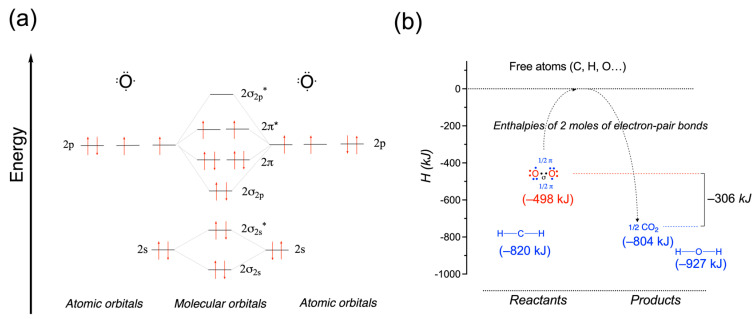
Structure of the O_2_ molecule. (**a**) Molecular orbitals in the ground state triplet O_2_ molecule formed from 2*s* and 2*p* atomic orbitals. The two electrons in the 2π* molecular orbital have opposite spin values. In the excited singlet O_2_ molecule, these two electrons have opposite spin values. (**b**) Bond formation enthalpies of two moles of electron-pair bonds in O_2_ and CO_2_. The bond formation enthalpies are lower (more negative) for stronger bonds [8]. O_2_ is represented in the ground state of the diradical dioxygen molecule with the two unpaired electrons participating in the π bonding (strong bond due to resonance stabilization: −418 kJ mol^−1^) and one weak O—O σ bond (79 kJ mol^−1^ vs. −204 kJ mol^−1^ in HO—OH). π electrons are shown in blue, while σ electrons are in black (From [1]). (*, non-bonding molecular orbitals).

The triplet diradical state of O_2_ (two unpaired electrons with parallel aligned spins, Figure 5a) results in spin restrictions making reactions with molecules in the singlet state (all electrons are paired with opposite spins) very slowly, even when they are thermodynamically favorable. Put differently, though highly abundant in our atmosphere, O_2_ is unreactive against other molecules in reactions that are thermodynamically exothermic (hydrogen abstraction, for instance). Reactions of O_2_ with organic molecules require strongly lowering the activation energy by transforming triplet to singlet O_2_—its first electronically excited state [35]. Under conditions compatible with life, this can only be performed by means of enzymes, such as oxidases [36].

In the triplet state, O_2_ is a diradical held together with a binding energy of only 498 kJ mol^−1^, but the oxygen atom forms two strong bonds in each of its combustion products: C=O double bond in CO_2_ (2 × 804 kJ mol^−1^) and H_2_O (927 kJ mol^−1^). As a result, each mole of O_2_ releases, on average, 460 kJ mol^−1^ (Figure 5b). It is the particularly weak bond in the oxygen molecule that makes combustion one of the most exothermic reactions in chemistry. In contrast, the bonding strengths of organic molecules are very similar to those of combustion products [1,3].

How can these particular properties of the O_2_ molecule be explained? The σ bond of O_2_ is relatively weak (78 kJ mol^−1^ or 19 kcal/mol, footnote 57 in [34]), allowing for the formation of two molecules of H_2_O and the release of a large amount of energy [34]. This contrasts with most organic molecules, where the π bond is weaker than the σ bond. On the other hand, resonance stabilization of the π bond (418 kJ mol^−1^) is responsible for the low reactivity of O_2_ [34]. In other words, the weak σ bond is responsible for the thermodynamic instability of O_2_, while the strong π bond increases the activation energy for the reaction with other molecules.

Therefore, we can conclude that the energy released by combustion comes from the substitution of the weak O_2_ double bond by a stronger one and not from the particular “energy richness” of the reduced substrates (sugars, lipids, etc.) [8]. This led Schmidt-Rohr to consider O_2_ as an “energy-rich molecule” [8], in agreement with the chemical definition of this concept (Section 2.1). It thus appears that O_2_ is the molecule that stores most of the biochemical energy in large organisms. It is the major high-energy molecule in the biosphere.

## 4. The Concept of Transfer Potential

### 4.1. Group Transfer

A difficulty related to the concept of the “energy-rich” molecule is that limiting oneself to the difference in energy does not explain the coupling mechanism by which the energy released by ATP hydrolysis is used to drive an unfavorable (endergonic) process. It does not explain how this energy is transferred in biochemical processes [16]. Therefore, some authors introduced the concept of group transfer potential [12,37], referring to a compound that is kinetically stable, but once activated, has a high potential of transferring a group to a suitable acceptor. In addition to these chemical groups, the concept of transfer potential can be extended to acids, which are molecules with a high H^+^ transfer potential, and reductants, which have a high electron transfer potential (Table 3) [37]. Note that, in chemistry, the term group is used to designate a set of atoms that exhibits similar chemical properties regardless of the compound in which it appears.

As shown above, some phosphoanhydrides, thioesters, and dioxygen are high-energy molecules, but, in contrast to ATP, dioxygen does not transfer any group. Hence, we think that the notions of high-energy molecule and high group transfer potential are not equivalent, and the second cannot replace the first. This difference has also recently been illustrated by Schmidt-Rohr concerning the NAD(P)H molecule [3]. Indeed, if NAD(P)H is the reducing agent par excellence in biochemistry, the reduced form has a high electron transfer potential but is not “energy-rich” [15].

Therefore, we suggest treating the concepts of “energy-richness” and high group transfer potential as distinct; one comes from thermodynamics, the other from chemical kinetics based on the notion of a leaving group. In chemistry, a good leaving group is an electron-withdrawing group generating a stabilized ion (often the anion of a strong acid or its conjugate base) after breaking a covalent bond (heterolytic cleavage). Good electron-withdrawing groups increase the O—P bond length, increasing the energy of the reactant (Δ*G*° becomes more negative) and decreasing the energy of the transition state (decreasing the energy of activation) [37,38,39]. Generally, the detached group is stabilized by the solvent and by resonance, adding a thermodynamic dimension. This is exactly the case of molecules with high phosphate group transfer potential, in contrast to those with low transfer potential (Table 2).

### 4.2. Proton Transfer

The high phosphoryl group transfer potential of ATP (with water as acceptor) is illustrated by the highly negative Δ*G*′°, meaning that at equilibrium, *K*_eq_ >> 1. As far as H^+^ donors are concerned, a high H^+^ transfer potential is observed only with very strong mineral acids. Organic acids encountered in a biochemical environment are relatively weak acids with a p*K*_a_ between 3 and 5. Hence, for acetic acid with a p*K*_a_ of 4.8, we can easily calculate a Δ*G*° of +27 kJ mol^−1^ (Δ*G*° = -RT ln *K*_a_ and *K*_a_ = 10^−p*K*a^ = 10^−4.8^ = 1.58 × 10^−5^) under standard conditions ([H^+^] = 1 M) [40]. As expected, acetic acid does not have a high transfer potential for protons. On the other hand, for HCl which completely dissociates in aqueous medium, the Δ*G*° would be very negative.

### 4.3. Electron Transfer: Are Reduced Molecules Rich in Energy?

The standard presentation of energy-yielding reactions in most biochemistry texts suggests that the bulk of the required energy to produce ATP comes from NADH. It is also implied that NADPH is the energy-carrying molecule produced in the first stage of photosynthesis, and that it provides the energy to fuel the carbon assimilation in the second stage. There are several reasons why reducing agents have been considered as sources of energy in metabolic as well as non-biological reactions. There are well-known observations, for instance, that highly reduced compounds such as n-alkanes are excellent fuels for thermal engines. This concept includes the notion that strong reducing agents have a high electron transfer potential.

A well-known example of an energy-yielding redox reaction is the sequence of reactions catalyzed by complex I in the respiratory chain:

NADH + H^+^ + Q → NAD^+^ + QH_2_              Δ*G*′° = −82 kJ mol^−1^

The reaction is exergonic and is coupled to transmembrane proton transport, yielding a proton-motive force. More generally, oxidative phosphorylation—which is the most important energy-yielding process in living organisms—is fueled by the oxidation (through the respiratory chain) of the reducing substrates NADH and FADH (succinate). It is thus believed that the latter are the energy-rich molecules that fuel the reaction sequence.

More generally, there is the idea that a compound having a high group transfer potential must be “energy-rich”. The following consideration, however, should convincingly show that neither hydrocarbons nor NADH are energy-rich molecules. For a more detailed discussion, see [15].

First, it must be emphasized that even methane (which is the most reduced hydrocarbon) does not release energy when it breaks up; the reaction is endothermic [15]:

CH_4_ → C + 2 H_2_.         Δ*G*° ≈ 50 kJ mol^−1^

This is consistent with the stability of the CH_4_ molecule containing only relatively strong C—H bonds. Only the combustion of methane is strongly exergonic. As previously discussed, this is linked to the fact that the O_2_ molecules, not CH_4_, are energy-rich [8,15].

In addition, the analysis of the energetics of the reactions of NADH with various molecules (other than O_2_), shows that these reactions never yield high amounts of energy (some of them are actually endergonic). For instance, the last reaction of lactic acid fermentation has a Δ*G*′° of only −25 kJ mol^−1^:

NADH + H^+^ + pyruvate → NAD^+^ + lactate     Δ*G*′° = −25 kJ mol^−1^

This and many other examples show that NAD(P)H does not qualify as a high-energy molecule. How can this view be reconciled with the fact that NADH has a high electron transfer potential? In fact, changes in redox state are accompanied by an instant reorganization of molecular orbitals in such a way that the oxidized form is associated with a free orbital of particularly low energy, while the reduced form is associated with an occupied orbital of particularly high energy. Therefore, these molecules have a natural tendency for the oxidized form to accept electrons and for the reduced form to donate electrons [1,41].

The highly exergonic character of the reaction sequence in the respiratory chain is linked to the energy-rich character of O_2_ [15]. With respect to this, it is interesting to note that the energy-rich O_2_ is the most oxidized form of oxygen, while H_2_O is the most reduced one, again refuting the idea that reduced molecules are the energy-rich forms.

## 5. Chemical Coupling Through Phosphoryl Group Transfer

As pointed out by Klotz [37], in order to compare group transfer potentials of the donor molecules, it is convenient to select a standard acceptor molecule, which is usually H_2_O: the more the free energy of hydrolysis of a phosphate bond is negative, the higher is its phosphoryl group transfer potential. Quantitatively, this notion was defined as numerically equal to −Δ*G*′° (Table 2).

**Figure 6:**
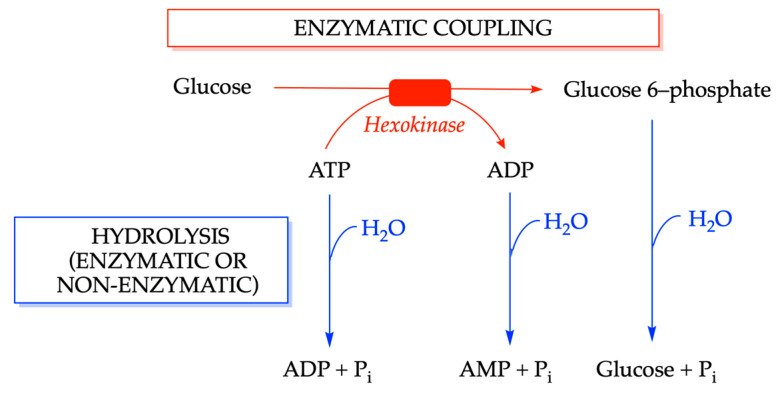
Enzymatic coupling versus hydrolysis. Though thermodynamically favorable, in the absence of enzyme, the formation of glucose 6-phosphate from glucose and ATP will never occur, because it requires the recognition of two substrates and their correct adjustment for the transfer of the phosphoryl group from the donor to the acceptor.

However, simple hydrolysis of ATP hardly ever happens in living cells. Like O_2_, ATP, while thermodynamically unstable, is relatively stable from a kinetic point of view. If ATP hydrolysis occurred in isolation, it would simply increase the temperature of the system, an inefficient way to speed up favorable reactions and to drive unfavorable processes. The reverse reaction invariably involves the abduction of a hydrogen (H) and hydroxyl (OH) group to form water (H_2_O) and is therefore called “dehydrating” condensations (Figure 4b). These reactions cannot occur in aqueous solutions as the abundance of water would favor the reverse hydrolytic reaction (Figure 6).

However, in most cases, the phosphoryl group is not transferred to water, but to an organic molecule. Each phosphorylated compound is able to transfer a phosphoryl group to an acceptor lower down the scale, provided that a coupling mechanism is available. (Note that a simple reversal of the reaction changes the donor and acceptor molecules.)

The most obvious coupling reaction would be the transfer of a phosphoryl group from ATP to an acceptor molecule to form a phosphoester as catalyzed by phosphotransferases (kinases): ATP + X-OH ⇄ ADP + X-O-P. An example of such a reaction is given by the hexokinase-catalyzed synthesis of glucose 6-phosphate:

Glucose + ATP → glucose 6-phosphate + ADP

The ΔG′° for this reaction is −16.7 kJ mol^−1^, much less from the −30.5 kJ mol^−1^ for ATP hydrolysis (transfer to water). Phosphorylation of glucose by ATP, though thermodynamically favorable, never occurs at a measurable rate in the absence of a catalyst. Upon binding of glucose, hexokinase undergoes an induced-fit conformational change excluding water from the active site, preventing ATP hydrolysis [2]. Subsequently, a phosphoryl group is then transferred from ATP to glucose at the active site of the enzyme. Thus, hexokinase, like many other enzymes involved in energetic coupling, is an energy transducer (or molecular machine) rather than a mere catalyst.

The concepts of coupling and hydrolysis are illustrated in Figure 6. The change in the standard free enthalpy Δ*G*′° when the terminal phosphoryl group is transferred from ATP to H_2_O is about −30.5 kJ mol^−1^. On the other hand, when glucose 6-phosphate transfers its phosphoryl group to H_2_O, Δ*G*′° is about −13.8 kJ mol^−1^. Thus, ATP has a substantially greater phosphoryl transfer potential than glucose 6-phosphate. Now if we write the two simultaneous reactions (ATP hydrolysis and glucose phosphorylation) and we know the Δ*G*′° values for the two reactions, we can readily estimate ΔG′° for the global reaction with good approximation [42]:

ATP + H_2_O     →     ADP + P_i_                     Δ*G*′° = −30.5 kJ mol^−1^

glucose + P_i_     →     glucose 6-phosphate + H_2_O   Δ*G*′° = 13.8 kJ mol^−1^

____________________________________________________________________

ATP + glucose → glucose 6-phosphate + ADP         Δ*G*′° = −16.7 kJ mol^−1^

**Figure 7:**
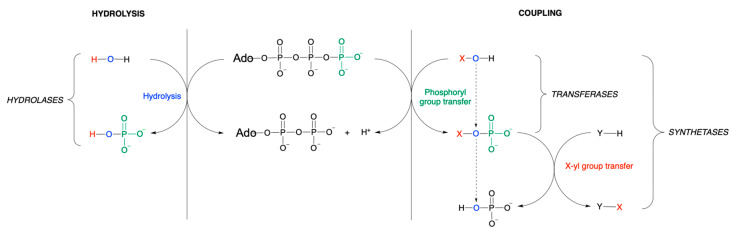
Hydrolysis and nucleophile (X—O—H)-dependent phosphoryl group transfer. In hydrolase (EC 3…) catalyzed reactions and in the absence of coupling, the free energy is dissipated as heat (see also Figure 6). In transfer reactions, the terminal phosphoryl group of ATP is transferred on X—OH, forming X-yl phosphate and ADP. In a simple transfer mechanism catalyzed by transferases (EC 2…), the reaction stops here. In synthetase catalyzed reactions (ligases, EC 6…), the X-yl group is, in a second step, transferred on Y—H releasing P_i_. Note that the oxygen of the nucleophile is transferred to the phosphoryl group and ends up in inorganic phosphate, and as expressed by de Duve [44], “mimics the cryptic path of water in a hydrolytic reaction” (modified from [44]).

Such thermodynamic calculations using Gibbs free energy yield correct values for the Δ*G*′° of the global reaction. However, it is quite misleading when we consider the mechanism of energy coupling, as the latter does not involve the hydrolysis of ATP. Neither H_2_O nor P_i_ appear in the global reaction or in the enzyme mechanism (Figure 7).

De Duve used the formulation of a "cryptic journey of “water” from X-O-H to the phosphate through the intermediate X-O-P" [43]. Nevertheless, it is highly questionable to use the free energy of the hydrolysis of ATP in such reactions [20].

Indeed, in many classical biochemistry textbooks, it is stated that the hydrolysis of ATP is coupled to various endergonic reactions (for discussion see [6,18,20]). Most textbooks correctly highlight the role of enzymes as coupling agents in biochemical reactions that otherwise would not occur. Indeed, without considering an enzymatic reaction mechanism, the statement “ATP hydrolysis drives unfavorable processes” is an explanatory black box and should be avoided [18]. The confusion stems from the fact that the free energy of hydrolysis is presented as a measure to compare different energy-rich compounds (as we did in Table 2) but is useless from a mechanistic point of view.

In conclusion, if we focus solely on ATP hydrolysis, we miss out the mechanisms by which ATP drives thermodynamically unfavorable processes such as the phosphorylation of various substrates. It should also be pointed out that even in some ATP hydrolyzing molecular machines such as ion transport ATPases (Na-K- and Ca-ATPases), ATP also acts as a phosphorylating agent: phosphorylation of an aspartate residue induces a conformational change that plays a key role in ion translocation [44].

## 6. Coupling of Two Reactions with ATP Hydrolysis (Ligase-Catalyzed Reactions)

In a different kind of reaction, the energy of the O-P bond of ATP is used to couple two molecules X and Y: ATP + X-OH + Y-H ⇄ ADP + P_i_ + X-Y (Figure 7). This would be the typical reaction catalyzed by a synthetase (ligase) such a glutamine synthetase:

L-glutamate + ATP + NH_3_ ⇄ γ-glutamyl phosphate + ADP + NH_3_ ⇄ L-glutamine + P_i_

Such reactions are sometimes written using two coupled half-reactions:

L-glutamate + H^+^ + NH_3_ → L-glutamine + H_2_O

ATP + H_2_O → ADP + P_i_

Once again, this would suggest that ATP is actually hydrolyzed, which is not the case and no H_2_O molecule is involved in the reaction mechanism [45], but instead the oxygen atom of the carboxyl group of glutamate (X–O–H) plays the role of the oxygen atom of water in a hydrolysis reaction (Figure 7).

**Figure 8:**
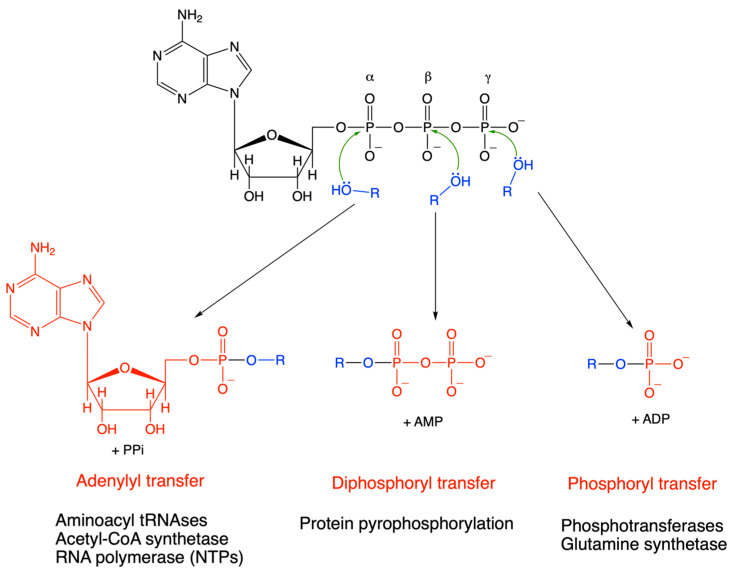
Group transfer reactions from ATP. The nucleophile can attack either the α, β, or γ phosphorus. In the first case, an adenylyl or, more generally, a nucleotidyl group is transferred, for instance, during synthesis of nucleic acids, the reactions catalyzed by aminoacyl tRNAses or acetyl-CoA synthetase. In the second case, a pyrophosphoryl group is transferred and in the third case, a phosphoryl group.

Note that most biosynthetic reactions rely on the scission of the α—β phosphoanhydride bond in ATP (and other nucleoside triphosphates), leading to the cleavage (for instance, the condensation of two amino acids during protein synthesis or the formation of a phosphodiester bond during nucleic acid synthesis) or the transfer of a diphosphoryl group (for instance, protein pyrophosphorylation [46]) (Figure 8). This is justified by the higher group transfer potential of the α—β compared to the β—γ phosphoanhydride bond (Table 2). In some cases, a nucleotidyl group (transnucleotidylation) is transferred, but the principle remains the same.

In the latter case, the transfer of a nucleotidyl group leads to the release of pyrophosphate which is rapidly hydrolyzed by omnipresent pyrophophatases. This highly exergonic reaction (−33 kJ mol^−1^, Table 2) renders many biosynthetic reactions irreversible.

## 7. Substrate-Level Phosphorylation and Thioesters

**Figure 9:**
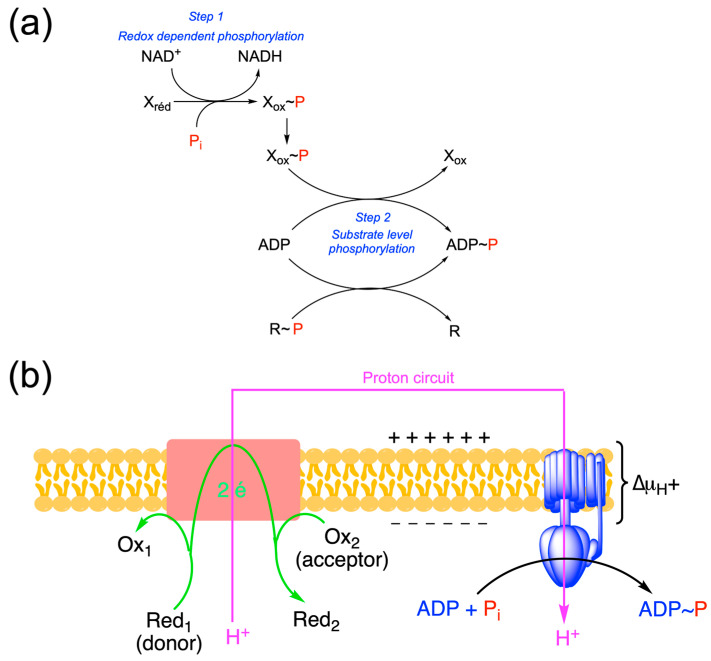
The two mechanisms of ATP synthesis. (**a**) Substrate-level phosphorylation. Redox-dependent substrate-level phosphorylation requires two steps. The first step consists of the redox-dependent synthesis of an intermediate with a high phosphoryl group transfer potential (X_ox_~P). The energy comes from the oxidation of X_red_ to X_ox_. In a second step, the phosphoryl group is transferred to ADP by a phosphotransferase (for instance phosphoglycerate kinase, EC 2.7.2.3). Alternatively, substrate-level phosphorylation can occur without the prior redox-dependent step from a high phosphoryl group donor (R~P, for instance, phosphoenolpyruvate, creatine phosphate…). In the latter case, the phosphorylation most often consists of P_i_ recycling as donor will be regenerated through another molecule of ATP (see Figure 10b) (**b**) ATP synthesis by chemiosmotic coupling. In this process, the energy also comes from the oxidation of a reductant, but there is no phosphorylated intermediate.

ATP can be generated by two processes: phosphorylation at the substrate level and chemiosmotic coupling (Figure 9). These two mechanisms probably have already coexisted in the Last Universal Common Ancestor (LUCA). Indeed, phylogenetic analyses suggest the existence of a rotating ATP synthase in LUCA. It also seems that phosphorylation at the substrate level was present in LUCA and would have preceded chemiosmotic coupling [7].

Substrate-level phosphorylation consists of the synthesis of a high-potential intermediate (acyl phosphate, enol phosphate or thioester) that, when hydrolyzed, provides free energy for the synthesis of ATP via a coupling mechanism (Figure 9a step 2):

R ~ P + ADP → R + ATP

Acyl phosphates and enol phosphates have a higher phosphoryl group transfer potential than ATP, which allows the reaction to proceed toward the synthesis of the latter (Table 2).

Thioesters play a particularly important role in substrate-level phosphorylation. Hence, in the TCA cycle, the reaction catalyzed by succcinyl-CoA synthetase uses the energy from the hydrolysis of the thioester succinyl-CoA, for the synthesis of GTP or ATP:

Succinyl-CoA + GDP (ADP) + P_i_   →   succinate + GTP (ATP)    Δ*G*′° = −3.3 kJ mol^−1^ (Succinyl-CoA synthetase, EC 6.2.1.4)

Other reactions involving thioester-dependent ATP synthesis are found in certain bacteria [47]:

Acetyl-CoA + P_i_     →     acetyl phosphate + CoA   Δ*G*’° = 11 kJ mol^−1^

(Phosphate acetyltransferase, EC 2.3.1.8)

Acetyl phosphate can then be used for the synthesis of ATP through the reaction:

Acetyl phosphate + ADP   →   acetate + ATP        Δ*G*’° = −12 kJ mol^−1^ (Acetate kinase, EC 2.7.2.1)

In another type of reaction, the thioester is a catalytic intermediate. Among these reactions, probably the most important is the sequence catalyzed by glyceraldehyde-3P dehydrogenase and phosphoglycerate kinase in glycolysis:

Glyceraldehyde 3-phosphate + NAD^+^ + ADP + P_i_  →

3-phosphoglycerate + NADH + H^+^ + ATP    Δ*G*′° = −12.3 kJ mol^−1^

**Figure 10:**
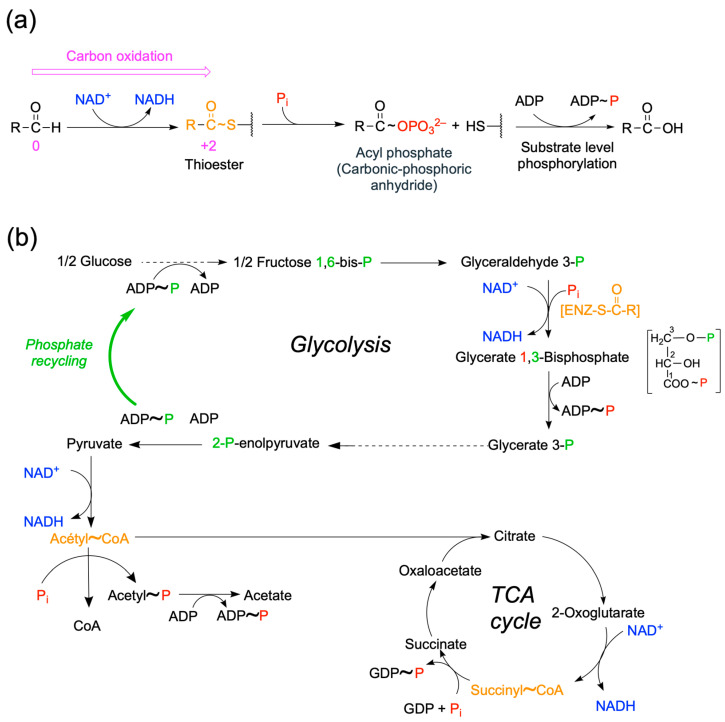
Importance of thioesters in ATP synthesis. (**a**) General mechanism of coupling the formation of a thioester to ATP synthesis as occurring in glyceraldehyde-3-phosphate dehydrogenase (EC 1.2.1.12). The first step corresponds to the redox-dependent formation of a thioester intermediate on the enzyme and the second to the formation of 1,3-bisphosphoglycerate. The oxidation numbers of the carbon are indicated in magenta. The high potential phosphoryl group product (Table 2) can then serve as substrate for the phosphorylation of ADP to ATP. (**b**) Substrate-level phosphorylation in glycolysis and TCA cycle. The phosphates (P_i_) incorporated by thioester-dependent mechanisms are indicated in red. Thioesters (in yellow) are formed by redox-dependent mechanisms catalyzed either by glyceraldehyde-3-phosphate, pyruvate, or oxoglutarate dehydrogenases. Phosphates recycled via ATP (ADP~P) are represented in green.

In this reaction sequence, the key step is the synthesis of 1,3-bisphosphoglycerate from glyceraldehyde 3-phosphate. This reaction is driven by the exergonic oxidation of the aldehyde to a carboxyl by NAD^+^, coupled to the formation of the energy-rich thioester in the active site (Figure 10a). The next step is the binding of inorganic phosphate to the thioester, forming an acyl phosphate intermediate, 1,3-bisphosphoglycerate. The high phosphoryl group transfer potential of the latter is then used to generate ATP.

This reaction is of paramount importance because it allows direct entry of phosphate into the cellular metabolism, in contrast to the reaction catalyzed for instance by pyruvate kinase during the last step of glycolysis (Figure 10b), where the phosphoryl group of phosphoenolpyruvate is transferred from ADP to ATP. It is evident from the latter reaction that the phosphate transferred to fructose 1,6-bisphosphate from ATP in the early stages of glycolysis is just recycled.

Remarkably, in most substrate-level phosphorylation reactions, the energy comes from the breakdown of a thioester (either acetyl-CoA or succinyl-CoA) or a protein-bound intermediate formed in the reaction catalyzed by glyceraldehyde 3-phosphate dehydrogenase. This point has been widely discussed by Christian de Duve, who gave thioesters a fundamental role in the appearance of life [48]. This is possible because the free energy of hydrolysis of a thioester is very negative compared to that of an O-ester (Section 3.3 and Table 2).

## 8. Conclusions

From the above considerations, it follows that we can distinguish between three chemically distinct groups of energy-rich molecules, phosphorylated molecules (phosphoanhydrides, enol phosphates, acyl phosphates, and guanidine phosphates), and acyl-thioesters (mainly acetyl-CoA and succinyl-CoA) as well as O_2_. They have in common that they are thermodynamically unstable, while being kinetically stable. Thermodynamic instability, because of low bonding energy, is a prerequisite. The molecule must possess a relative kinetic stability (high activation energy) in order to not react with neighboring alien molecules. This entails that their reactivity is dependent on the presence of catalysts (enzymes) that allow the reactions to occur under physiological conditions and simultaneously allow a circumstantial allosteric control.

As we have amply discussed, the term “energy-rich” molecule is an ambiguous expression in the sense that these molecules contain at least one low energy molecular bond that, in exchange for a high-energy molecular bond, leads a release in energy. To lift this apparent contradiction, it was proposed to replace the term “energy-rich” molecule by “high group transfer potential”. While these two notions are more or less equivalent for organic molecules such as ATP or acetyl-CoA, the O_2_ molecule is an intruder in the sense that, while “energy-rich”, it has no transfer potential (Table 4). Moreover, as has been convincingly shown by Schmidt-Rohr [15] that reduced molecules are not necessarily energy-rich, though they may have a high electron transferring potential.

If hexoses and fatty acids are neither energy-rich nor have a high electron transfer potential, then one can wonder the following: what do they actually store? An answer to that question could be that fuel molecules store potential reducing power. During catabolism, molecular bond rearrangements in the fuel molecules lead to the oxidation of their carbon atoms, powering an electron flow towards NAD(P)H that can then drive (O_2_-dependent) ATP synthesis and biosynthetic processes. Such a view clearly justifies the separation of electron flow from energy flow as shown in Figure 2.

Another important point concerns the mechanisms of coupling. The free energy of hydrolysis is most often used as a measure of the maximum energy that can be transferred during coupling events. Here, the term hydrolysis forcibly implies that the acceptor is H_2_O. However, in most coupling reactions, H_2_O is totally absent as its presence even might prevent coupling (Figure 6). Hence, the free energy of hydrolysis is a useful indicator of the group transfer potential of a molecule, but it is not relevant from a mechanistic point of view.

While ATP is the main donor for phosphoryl group donor, acetyl-CoA is the donor for acetyl groups and also plays an important role as coupling agent between catabolism and anabolism (Figure 1).

Hence, the reducing power is the main driving force for ATP synthesis both in substrate-level and oxidative phosphorylation. Other transphosphorylation reactions, such as those catalyzed for instance by creatine or pyruvate kinase, only recycle ATP and P_i_, the synthesis of creatine phosphate, and phosphoenolpyruvate depending, directly or indirectly, on ATP (Figure 10b).

It is true that the energy released from glucose degradation (glycolysis) in the absence of O_2_ is sufficient to sustain the life of some anaerobic organisms. But evidently, O_2_ is the required energy source for complex multicellular life, especially animals [15]. The presence of sufficient amounts of O_2_ in the atmosphere allowed the emergence of large complex organisms with higher energy needs.

The emergence of eukaryotic cells goes hand in hand with an internalization of specific structures such as mitochondria and chloroplasts, specialized in the synthesis of ATP by a chemiosmotic mechanism and the genesis of a proton gradient. This is a revolution that frees up the cell membrane for other types of ion gradients. Thus, the generation of electrical signals (action potential) essential to multicellular life is decoupled from the energy function, though the latter still feeds the establishment and maintenance of ion gradients across the plasma membrane.

## Figures and Tables

**Table 1:** Average bond dissociation enthalpies (Δ_f_*H*°) for some molecular bonds in common organic molecules [8,9,10]. Δ_f_*H*° depends on the molecule and exact values may differ between sources.

Bond	Δ_f_*H*° (kJ mol^−1^)
C—C	−346
C=C	−615
C—H	−413
C—O	−351
C=O (in CO_2_)	−804
C—N	−305
C—S	−259
PO—P	~334 ^1^
O=O	−498
O—H	−463
O—O (H_2_O_2_)	−139

^1^ See reference [11].

**Table 3:** Comparison between different kinds of transfer potentials.

	Group Transfer Potential (NTPs or Thioesters)	Electron Transfer Potential (Redox Potential)	Proton Transfer Potential (Acidity)
Equation	A–O~**P** → A + P_i_	A → A^+^ + e^−^	AH → A^−^ + H^+^
Equation with acceptor	A–O~**P** + H_2_O → A-OH + P_i_ + H^+^	A + H^+^ → A^+^ + ½ H_2_	AH + H_2_O →A^−^ + H_3_O^+^
Measure of transfer potential (by hydrolysis)	Δ_h_*G*° = Δ*G*°	$\varepsilon {}^{\circ}=\frac{-\Delta G{}^{\circ}}{nF}$	${pK}_{a}=\frac{\Delta G{}^{\circ}}{2.303\;RT}$
Nature of the transfer potential	∝ Δ*G*° per mol of P_i_ transferred	∝ Δ*G*° per mol of e^−^ transferred	∝ Δ*G*° per mol of H^+^ transferred

From [1] and modified according to Klotz 1986 [37].

**Table 4:** High-energy molecules vs. molecules with high transfer potential (group, electrons, H^+^). Here, the term “group” refers only to functional groups, i.e., groups of atoms with distinctive properties linked to the rest of the molecule by a covalent bond (phosphoryl, acetyl…).

Molecule	High-Energy Molecule	High Group Transfer Potential	Electron Transfer Potential	H^+^ Transfer Potential
NTP	Yes	Yes (phosphoryl)	No	No
Acetyl-CoA	Yes	Yes (acetyl)	No	No
O_2_	Yes	No	No	No
Hexoses, fatty acids	No	No	No ^1^	No
NAD(P)H	No	No	Yes	No
AH (strong acids)	No	No	No	Yes

^1^ While the glucose molecule is readily oxidized to glucuronic acid, most of the reducing power of hexoses and fatty acids must be unlocked by metabolic transformations under biochemical conditions.

## Data Availability

No new data were created or analyzed in this study. Data sharing is not applicable to this article.

## Abbreviations

The following abbreviations are used in this manuscript:

TCA cycle	Tricarboxylic acid cycle

## References

[1] Bettendorff L. (2022). Reduced Nucleotides, Thiols and O2 in Cellular Redox Balance: A Biochemist’s View. Antioxidants.

[2] Voet D., Voet J.G., Pratt C.W. (2013). Principles of Biochemistry.

[3] Campbell M.K., Farrell S.O. (2009). Biochemistry.

[4] Alberty R.A., Cornish-Bowden A., Goldberg R.N., Hammes G.G., Tipton K., Westerhoff H.V. (2011). Recommendations for Terminology and Databases for Biochemical Thermodynamics. Biophys. Chem..

[5] Nath S. (2016). The Thermodynamic Efficiency of ATP Synthesis in Oxidative Phosphorylation. Biophys. Chem..

[6] Hapkiewicz A. (1991). Clarifying Chemical Bonding. Overcoming Our Misconceptions. Sci. Teach..

[7] Heitler W., London F. (1927). Wechselwirkung neutraler Atome und homöopolare Bindung nach der Quantenmechanik. Z. Physik.

[8] Schmidt-Rohr K. (2015). Why Combustions Are Always Exothermic, Yielding about 418 kJ per Mole of O_2_. J. Chem. Educ..

[9] Blackman A. (2014). Aylward and Findlay’s SI Chemical Data 7E.

[10] Brown T.L., LeMAy H.E., Bursten B.E. (1994). Chemistry: The Central Science.

[11] George P., Rutman R.J. (1960). The “High Energy Phosphate Bond” Concept. Prog. Biophys. Mol. Biol..

[12] Lipmann F. (1941). Metabolic Generation and Utilization of Phosphate Bond Energy. Advances in Enzymology—And Related Areas of Molecular Biology.

[13] Thornton W.M. (1917). XV. The Relation of Oxygen to the Heat of Combustion of Organic Compounds. Lond. Edinb. Dublin Philos. Mag. J. Sci..

[14] Weiss H.M. (2008). Appreciating Oxygen. J. Chem. Educ..

[15] Schmidt-Rohr K. (2020). Oxygen Is the High-Energy Molecule Powering Complex Multicellular Life: Fundamental Corrections to Traditional Bioenergetics. ACS Omega.

[16] Podolsky R.J., Morales M.F. (1956). The Enthalpy Change of Adenosine Triphosphate Hydrolysis. J. Biol. Chem..

[17] Atkinson D.E. (1977). Cellular Energy Metabolism and Its Regulation.

[18] Franovic C.G.-C., Williams N.R., Noyes K., Klymkowsky M.W., Cooper M.M. (2023). How Do Instructors Explain The Mechanism by Which ATP Drives Unfavorable Processes?. LSE.

[19] Green A.I., Parent K.N., Underwood S.M., Matz R.L. (2021). Connecting Ideas Across Courses: Relating Energy, Bonds & How ATP Hydrolysis Powers a Molecular Motor. Am. Biol. Teach..

[20] Fontecilla-Camps J.C. (2022). The Complex Roles of Adenosine Triphosphate in Bioenergetics. Chembiochem.

[21] Ruben E.A., Plumley J.A., Chapman M.S., Evanseck J.D. (2008). Anomeric Effect in “High Energy” Phosphate Bonds. Selective Destabilization of the Scissile Bond and Modulation of the Exothermicity of Hydrolysis. J. Am. Chem. Soc..

[22] Pinna S., Kunz C., Halpern A., Harrison S.A., Jordan S.F., Ward J., Werner F., Lane N. (2022). A Prebiotic Basis for ATP as the Universal Energy Currency. PLoS Biol..

[23] Roy D., Najafian K., von Ragué Schleyer P. (2007). Chemical Evolution: The Mechanism of the Formation of Adenine under Prebiotic Conditions. Proc. Natl. Acad. Sci. USA.

[24] Banfalvi G. (2006). Why Ribose Was Selected as the Sugar Component of Nucleic Acids. DNA Cell Biol..

[25] Jencks W.P., Fasman G.D. (1976). Handbook of Biochemistry and Molecular Biology (Volume I).

[26] Frey P.A., Arabshahi A. (1995). Standard Free Energy Change for the Hydrolysis of the Alpha, Beta-Phosphoanhydride Bridge in ATP. Biochemistry.

[27] Colvin M.E., Evleth E., Akacem Y. (1995). Quantum Chemical Studies of Pyrophosphate Hydrolysis. J. Am. Chem. Soc..

[28] Kornberg A., Rao N.N., Ault-Riché D. (1999). Inorganic Polyphosphate: A Molecule of Many Functions. Annu. Rev. Biochem..

[29] Börner A., Zeidler J. (2023). The Chemistry of Biology: Basis and Origin of Evolution.

[30] Nelson D.L., Cox M.M. (2000). Lehninger Principles of Biochemistry.

[31] Yang W., Drueckhammer D.G. (2001). Understanding the Relative Acyl-Transfer Reactivity of Oxoesters and Thioesters:  Computational Analysis of Transition State Delocalization Effects. J. Am. Chem. Soc..

[32] De Duve C. (2005). Singularities: Landmarks on the Pathways of Life.

[33] Stryer L. (1995). Biochemsitry.

[34] Borden W.T., Hoffmann R., Stuyver T., Chen B. (2017). Dioxygen: What Makes This Triplet Diradical Kinetically Persistent?. J. Am. Chem. Soc..

[35] Mondal S., Nguyen H.T.K., Hauschild R., Freunberger S.A. (2025). Marcus Kinetics Control Singlet and Triplet Oxygen Evolving from Superoxide. Nature.

[36] Minaev B.P., Sakhno T.V., Panchenko O.O., Sakhno Y.E. (2024). Spin-Orbit Interaction During the Activation of Molecular Oxygen by Oxidases and Cofactor-Free Oxygenases: A Review. Theor. Exp. Chem..

[37] Klotz I.M. (1986). Introduction to Biomolecular Energetics.

[38] Kirby A.J., Nome F. (2015). Fundamentals of Phosphate Transfer. Acc. Chem. Res..

[39] Mulashkina T.I., Kulakova A.M., Nemukhin A.V., Khrenova M.G. (2024). Comparison of the Mechanisms of Hydrolysis of Organophosphates with Good and Poor Leaving Group by Phosphotriesterase from Pseudomonas Diminuta. Russ. J. Phys. Chem. A.

[40] Metzler D.E. (2001). Biochemistry. The Chemical Reactions of Living Cells.

[41] Pullman B., Pullman A. (1959). The Electronic Structure of the Respiratory Coenzymes. Proc. Natl. Acad. Sci. USA.

[42] Meurer F., Bobrownik M., Sadowski G., Held C. (2016). Standard Gibbs Energy of Metabolic Reactions: I. Hexokinase Reaction. Biochemistry.

[43] De Duve C. (2005). Singularités—Jalons Sur Les Chemins de La Vie.

[44] Nguyen P.T., Deisl C., Fine M., Tippetts T.S., Uchikawa E., Bai X., Levine B. (2022). Structural Basis for Gating Mechanism of the Human Sodium-Potassium Pump. Nat. Commun..

[45] Eisenberg D., Gill H.S., Pfluegl G.M., Rotstein S.H. (2000). Structure-Function Relationships of Glutamine Synthetases. Biochim. Biophys. Acta.

[46] Morgan J.A.M., Singh A., Kurz L., Nadler-Holly M., Ruwolt M., Ganguli S., Sharma S., Penkert M., Krause E., Liu F. (2024). Extensive Protein Pyrophosphorylation Revealed in Human Cell Lines. Nat. Chem. Biol..

[47] Martin W.F. (2020). Older Than Genes: The Acetyl CoA Pathway and Origins. Front. Microbiol..

[48] De Duve C. (1990). Construire Une Cellule. Essai Sur La Nature et l’origine de La Vie.

